# Our Enterprize

**Published:** 1833

**Authors:** 


					﻿3. Spontaneous Poisonous Changes in Bread and Meat.—M.
Ciievallier has published a paper in the Journ. de Chem. Med.
on certain changes which bread and meat, long kept, are apt to
undergo, by which they are rendered poisonous. The follow-
ing case occurred in the practice of M. Bricheteau, in August,
1831. A woman, aged about 40, purchased of a pork butcher
and eat through the day, some slices of bacon. In the evening
M. Bricheteau found that she had been vomiting for several
hours.
“The abdomen was excessively tender. She had frequent stools,
with tenesmus, and she complained of general pain. Cataplasms
were applied to the abdomen, and she was ordered lavements, diluent
drinks, and absolute diet. Notwithstanding this treatment, the patient
had that night above fifty stools, and the abdominal pains continued
very severe. Leeches were consequently applied, a warm bath or-
dered, and the previous treatment continued. In two days the patient
recovered. These symptoms at such a time might have readily been
attributed to other causes, had not a young woman, who had eaten a
very small morsel of the same meat, experienced analagous accidents.
And it further appeared that a third person had been very ill after
eating pork purchased at the same time and place.
On the 30th of May, 1832, we were directed to institute an official’
inquiry re pecting an occurrence of the same nature, and which gave
rise to the subjoined report:”
In May, 1832, a similar accident happened to another family,
apparently from a like cause. A commission, consisting of
MM. Durocher, Geeury and Chevallier, was directed to inquire
into the circumstances of the case. The following is the result
of their inquiry:
a Examination of the Meat.—The meat, a part of which had occa<-
sioned the illness of the female, was composed of several pieces cut
from a lump of a preparation known in the pork trade by the name of
Italian cheese, made of mixed fragments, strongly seasoned, and
converted into a kind of compact pie, which is sold in slices.- The
pieces we examined were covered, some with blue, and others with
green mould, the latter circumstance occasioning a copery appear-
ance. Having divided a portion into three parts, one was treated
with distilled water, and the solution tested by reagents, which proved
the absence of any poisonous metal. Another part was treated with
distilled water acidulated with nitric acid; the solution thus obtained
was evaporated, the residuum redissolved in water, and tested by
reagents, which, as before, gave no indication of any known poison..
The last part of the meat was introduced into a new crucible,, carbon-
ized and incinerated. The ashes did not contain the least traces of
copper. The same experiments repeated on the meat found at the
shop of the Sieur L., were attended with the same negative results.
From these facts it follows that the meat in question contained no
copper,but that it had undergone a marked alteration capable of pro;
ducing the accidents in question; nor is this the first example of poison]
ing by this particular substance. Dr. Paulus, of Saltz, has already
related the history of seven persons who became violently ill after
eating Italian cheese, and of whom three died. In 1824, a family
named Plagneard, at Paris, were also very dangerously affected after
partakinn of a ham pie which contained no metallic poison, but in
which the alteration in question had commenced.”
In the London Lancet, is contained an account of a case of
poisoning by mouldy bread. It occurred in the family of the
beadle of the parish of Hammersmith, Eng.
“ His wife purchased in the morning a loaf of bread, of which she ate
a slice at breakfast. Her son, 20 years of age, ate two slices of the
same bread toasted; almost immediately after the meal, both became
unwell, and diarrhoea, vomiting, and tenderness of the abdomen super-
vened, and several hours elapsed before these symptoms abated. The
loaf, a considerable portion of which we obtained, was of yellowish
color, though baked that morning, and heated for the ordinary length
of time. It was sprinkled over with minute fungiform vegetations,
the greater number of which were black, a few green, and several
yellow. It was soft, wet, inelastic, and so tough that it could be drawn
into strings. Its taste was unpleasant, its smell acrid, and it reddened
litmus paper when laid upon it.”
The mould, collected and eaten separately, was found to be
innoxious. It was ascertained by various experiments, that no
mineral poison was present.
“On analysis of the bread, it was found to contain the due propor-
tion of starch, amidine, sugar, and earthy substances, but the gluten
had undergone a marked alteration in its proportions.”
III. ORIGINAL.
rne western ^ournaL
E SYLVIS, ACQUE ATQ.UE AD SyLVAS NUNCIUS.
Cincinnati, June 30, 1833.
I. Our Enterprize.
The Editor begs leave to tender his acknowledgements to
the profession in the West, for the support that has enabled his
publisher to reach and finish the sixth volume of this work.
For the convenience of gentlemen who may now, or hereafter,
wish to subscribe, this volume will be made the first of a new
series or Hexade; its title page at once indicating it as volume
seventh of the entire work, and volume first of the second
Hexade.
The Western Journal is the oldest Medical, scientific, or lit-
erary periodical in the valley of the Mississippi, except news-
papers. It has struggled through various difficulties; and fal-
len successively into the hands of several publishers. Its pro-
jector and original Editor has been aided, at different times, by
two assistants, though at one period, to preserve its very exist-
ence, he alone, was both editor and publisher. His lust coad-
jutor was Dr. Finley, whose withdrawal must be regretted by
every reader of the Journal. All these changes were, perhaps,
in some degree inseparable from such an undertaking in a new
country. But the Editor has, as he believes, good reason for
anticipating a firmer and happier progress for the Journal here-
after, than that which it has passed through. Fie does not ex-
pect it to change publishers so often, as heretofore; he hopes to
see it issued with more punctuality, and flatters himself; that a
greater number of the respectable physicians of the West, will
hereafter inscribe on its pages the results of their experience.
To the East he does not venture to look either for patronage
or professional contribution. The Journal has never, yet, had
a dozen subscribers east of the mountains, nor have half that
number of his eastern brethren contributed to its pages! In
Philadelphia, the seat of his alma mater and the emporium of
the medical sciences, for some time the only subscriber was
the public spirited Matthew Carey, Esq., and when the learned
Dr. Bell, of that city, was writing the Treatise on Mineral Wa-
ters and Baths, which was lately commended to the readers of
this miscellany, he could not find a copy of it in the whole of
that great city, from which to transcribe the history of the prin-
cipal mineral springs of Kentucky and Ohio.
The Editor notes these facts, as items in the history of wes-
tern medical literature; and may add, that the periodical
press of the Eastern States, has seldom taken the slightest notice
of his Back-woods’ enterprize; although, with all its imperfec-
tions, it has received sufficient Back-woods’ patronage, to live
for a longer period, than a majority of the medical journals
that have shot forth in the East.
It will be the duty, as it is the desire and design, of the ori-
ginal, now the sole editor, to devote to it more time and atten-
tion, than he has heretofore done; and aided.by such of his pub-
lie and private pupils, scattered over the valley of the Mississip-
pi, as cultivate the profession, con amove, he hopes to render the
second Hexade, in all respects, superior to the first.
The plan of the work, admitting of Original Communications,
Reviews, and Miscellaneous Intelligence—analectic, analyti-
cal and original—has been devised, as best adapted to the dis-
semination of every kind of professional information, and fitted
to render the work Esylvis, acque atque ad sylvas, nuncius.
				

